# Induced Cooperation to Access a Shareable Reward Increases the Hierarchical Segregation of Wild Vervet Monkeys

**DOI:** 10.1371/journal.pone.0021993

**Published:** 2011-07-20

**Authors:** Riccardo Pansini

**Affiliations:** 1 Université de Strasbourg, Institut Pluridisciplinaire Hubert Curien, Strasbourg, France; 2 Éthologie Évolutive, CNRS, UMR7178, Strasbourg, France; 3 Loskop Dam Nature Reserve, Applied Behavioural Ecology and Ecosystem Research Unit, University of South Africa, Groblersdal, South Africa; University of Maribor, Slovenia

## Abstract

Until now cooperation experiments in primates have paid little attention to how cooperation can emerge and what effects are produced on the structure of a social group in nature. I performed field experiments with three groups of wild vervet monkeys in South Africa. I induced individuals to repeatedly approach and operate food containers. At least two individuals needed to operate the containers in order to get the reward. The recurrent partner associations observed before the experiment only partly predicted the forming of cooperative partnerships during the experiment. While most of the tested subjects cooperated with other partners, they preferred to do so with specific combinations of individuals and they tended not to mix with other group members outside these preferred partnerships. Cooperation therefore caused the relatively homogeneous networks I observed before the experiment to differentiate. Similar to a matching market, the food sharing partners selected each other limiting their choice. Interestingly neither sex nor age classes explained the specific partner matching. Kinship could not explain it either. Rather, higher ranking individuals cooperated with other higher ranking individuals, and lower ranking also matched among the same rank. This study reveals the key role dominance rank plays when food resources are patchy and can only be accessed through sharing with other individuals.

## Introduction

One of the key elements in evolution is the potential of individuals to act together in cooperation. Cooperation allows many individuals to achieve goals that can often not be accomplished by single individuals. Specifically, I define cooperation as any act jointly carried out so that there is a net gain for all individuals involved (following [1]). In mammals, events such as being able to identify feeding resources more easily and warn group members for predators are examples of evolutionary stable cooperation strategies.

After kin selection theory and the concept of inclusive fitness had proposed [2], the theory of the evolution of cooperation amongst unrelated individuals was further explained through reciprocal altruism. Reciprocal altruism focuses on the future benefit return of the cooperative act [3]. Being able to assess the outcome of repeated interaction is central when individuals can choose to cooperate or defect at turns among a range of partner options. The iteration of the cooperative act is a key element in the maintenance and stabilization of cooperation [4].

With this study, I am interested in why partners are chosen in relation to the investment required in the cooperative act (as formalised in biological market theories, [5], [6]). The choice individuals make to find suitable partners should be based on the quality of honest signals, indicating the qualities of potential partners. The evaluation of potential partner quality a posteriori can also occur through some sort of trial interaction. If cooperation with specific individuals does not produce a convenient outcome, partner switching should take place so to favour a search for the profitable combination of partners [7]. This perspective allows generalising further, because it takes into consideration the strategies accounted by multiple interacting individuals. Examples of animal societies applying multi-partner cooperation are many, but scant has been the specific analysis of these strategies under a game theoretical approach. The few, non-experimental models developed comprise lions defending their territories [8] and male dolphin alliances [9].

Following kin selection theory, animals living in a group are expected to cooperate taking into account kinship relationships and broad family bonds [2]. An example is provided by species of birds and mammals breeding cooperatively with multiple helpers attending the same nest (e.g. [10]). Cooperation in unrelated individuals, instead, may be rarer to observe even when the subjects belong to stable social groups (as recently reviewed by [11]).

Studying cooperation in any model species is of special concern when framed within the species' ecological context. Among other communal actions, accessing food as a group can be seen as a cooperative act that social species repeat several times on a daily basis. Communal food search should be a strategy worth to be adopted when the feeding resources are limited [12]. A relatively complex case of cooperation is food sharing. When it occurs, animals act together and make joint use of food resources that could in principle be used and monopolised by single individuals [13]. If cooperation is a stable strategy, food sharing is favoured over exclusive control over the resources.

In this study I induced wild vervet monkeys to cooperate in order to access to food. In my paradigm, the resources do not necessarily need to be offered by one individual to the other (as e.g. with offspring feeding by meerkats, [14]), but they are rather accessed by the animals at the same time (as with captive hyenas, [15] for experiments with captive rooks, where the resources are both offered and accessed at the same time, see [16]). I first analyse the ability of the tested subject to learn the cooperation task. Subsequently, I assess if the social network of the individuals modifies due to the induced cooperation. I did so by scoring how partners selected each other according to particular factors influencing their partner choice. Partner preferences should appear according to the individuals' choice to cooperate with specific group members as in a matching market [17].

The questions to investigate were: were preferred partners before and after the cooperation experiment the same, or did new combination of partners arise? Moreover, what were the factors inducing new combinations of individuals: sharing the same sex, the same age class, or similar rank? If the monkeys cooperate according to kin selection theory, the prediction is that they would combine taking into account relatedness.

The analysis of how preferred partnerships form is often missing in literature. This study represents a first step in answering this question and provides the first results concerning cooperative problem solving in primates in the field with experimental manipulation.

## Materials and Methods

### (a) Ethics Statement

My observations and experiments were performed in agreement with the guidelines for of the Association for the Study of Animal Behaviour. An ethical review permit was granted from the Applied Behavioural Ecology and Ecosystem Research Unit of the University of South Africa and a second national park permit was granted from the Mpumalanga Tourism and Park Association of South Africa.

### (b) Study subjects

I carried out this study in South Africa, 180 km northeast from Pretoria, at Loskop Dam Nature Reserve, in the Mpumalanga province. The reserve extends for 23,000 ha and consists mainly of ‘bushveld’ (some trees where the monkeys are most regularly found, thick acacia bushes and tall grasses). I studied three groups of wild vervet monkeys, *Chlorocebus aethiops*. Their social groups usually comprise an average of less than 20 individuals in Loskop Dam [18], [19], [20], but in other sites they can be more numerous [21]. The females are organised in a stable hierarchy, with mothers passing on their rank status to the offspring. Males instead migrate from group to group and their rank fluctuates. These social groups have a rough sex-ratio of 1.5 adult females against adult males [21].

The studied groups were: (1) the Picnic group with a total of 10 individuals (4 males and 6 females; 6 adults and 4 juveniles); (2) the Donga group with 19 individuals (8 males and 11 females; 11 adults and 8 juveniles); and (3) the Bay group with 17 individuals (11 males and 6 females; 10 adults and 7 juveniles. I define as juveniles as individuals of 4 years of age or less who have normally not bred yet. The infants younger than 1 year of age did not cooperated actively and were not included in the observations of this paper. They are therefore not listed in this demography. Their home ranges extended for about 1 km^2^ for each group. The Donga and the Bay group had adjacent home ranges; the Picnic group was at 6 km distance from the other two.

All three groups were habituated to human observers before the start of these experiments [18], [19], [20].

### (c) Outline of the experiment

After an initial observational period with the three groups, I started offering feeders to monkeys (for details on the feeding protocol and a video see Video S1 and Supporting Information S1). Similar to a reinforcement-based conditioning task, access to food was provided only when individuals would operate a push/pull button on top of the machines. This triggered the food release mechanism. I provided the feeders to the monkeys during several days. An experimental session or trial is defined as a day during which the feeders where provided to the monkeys. Two phases were implemented and followed to induce the monkeys to cooperate: (1) a training phase and (2) a cooperation phase.

**Figure 1:**
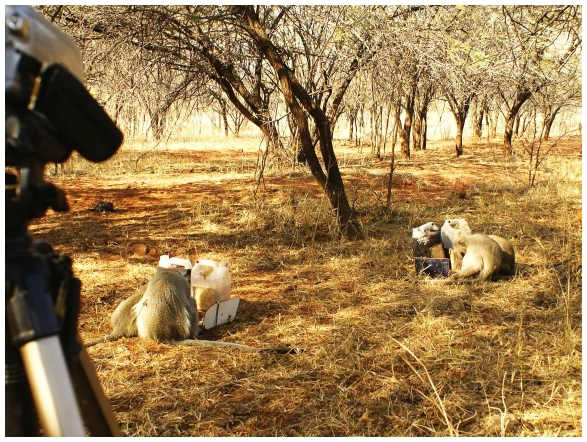
The setup of the experiment in the field. The picture was taken during the cooperation phase in July 2008 with the Picnic group. It shows two dyads of vervet monkeys cooperating and sharing food from the two joined feeders. The reward consisted of toasted rice cereal and was accessed by operating push/pull lever triggers on the top side of the boxes.

(1) The training phase was necessary to get the vervets used to the feeders and their functioning. The feeders were secured on the ground, and they could be accessed by one or more individuals indiscriminately. The individuals of a group were divided by me into two “cooperation-classes”: a small and a large cooperation-class. The smaller class was comprised of the same two individuals who became used having only access to black feeders. All the remaining individuals formed the larger class and learned that the only feeders functioning for them were coloured white (with the same shape and dimension of the black ones). The functioning of the correct feeder with the correct monkey class was possible with remote controls that activated and deactivated the push/pull trigger on top of the feeders. The two individuals of the smaller class were assigned and chosen so to be representative of the larger class and the entire group. The small class individuals thus had a predetermined combination of age, sex and rank position. They comprised one male and one female, one of these was adult and one juvenile, and one had a relatively high rank and the other a low rank.

(2) The cooperation phase was subsequently implemented. Couples of feeders, one black and one white, were now joined together (Figure 1). During this phase individuals of one class could not access the feeding resources unless waiting for the presence of members of the other class in front of the feeders. I therefore define cooperation in this specific experiment as the act of being at proximate distance and standing in front of the feeders by dyads or multiple partners. In the Supporting Information S1 I report data on normal foraging behaviour of these vervets. These data show that their foraging proximity distances were superior to the unnatural adjacent manifestations at the feeders.

Taking an economic perspective, the cooperation phase was designed to create a matching market [17], [22] whereby individuals coming from the mixed classes (at least one from the smaller and at least one from the larger) had to match and cooperate among each other in order to have access to food. The combinations possible at the feeders were limited in number and apparent from the asymmetric matrix made up by the two individuals of the smaller class joining with the individuals of the larger class. More importantly, and distinct from a traditional matching market, the individuals could combine with others, but at a following stage they could re-assort in other combinations.

The short side of the market was formed by the members of the smaller class as these individuals became in demand for cooperation (given their limited availability in number as compared to the larger cooperator class).

### (d) Behavioural data collection

The monkeys were observed during the daytime from 7 to 15 hours. Scan samples from all visible individuals (except the infants) were collected at 10 minutes intervals. In addition, an all-occurrence sampling technique was used. It consisted of the whole group being observed by more than one observer (with inter-rate consistency tested a priori). One observer (R. Pansini) continuously monitored the feeders, recorded all cooperation interactions and agonistic behaviours. At the same time, one or two assistant-observers recorded all-occurrence and scan behaviours of the rest of the monkeys not present at the feeders.

Recording of affiliative behaviours was done with all-occurrence sampling. The affiliative behaviours comprised allogrooming, contact sitting and social play. The agonistic interactions were collected to determine the relative rank of all individuals and consisted of recording all aggressive and submissive behaviour bouts started within 5 m radius from the feeders. Behavioural bouts were considered to have ended if these ceased for 5 or more seconds, replaced by another behaviour or a partner exchange. For each behavioural data point, the information recorded consisted of: (1) the starting time, allowing to infer the frequency of each bout (and not the duration in this case); (2) the time when the behaviour occurred – if before, during or after the experiment; (3) the identity of the individuals involved; (4) the direction of the behaviour when this was social (actor and recipient); (5) the distance place in relation to the feeders (when present) of where the bout took; (6) and the identity and the distance of the nearest neighbour individual (if present within 10 m distance).

The software Noldus Pocket Observer 2.1 and Pendragon Forms 5.1 were used for the collection of data in the field with Pocket PC's.

### (e) Statistics of association and interaction data and network structure

The several analyses produced are split in this section with roman numerals.

I use social network analysis to describe proximity and social relationships amongst the individuals. I define associations in terms of proximity distances; interactions, such as allogrooming are instead social behaviours exchanged by partners (following [23]).

For producing the statistics of association and interaction data and to structure the networks, I obtained (a) social differentiation indexes, (b) affiliation and cooperation rate indexes, and (c) standard errors of social differentiations.

i) The social differentiation index describes how varied the social system is [23]. It is an estimate of the coefficient of variation of the proportion of sampling periods dyads spend together, calculated by removing an estimate of the sampling variance from the coefficient of variation of the estimated association indices (calculated in the appendix of [24]). As a rule of thumb, Whitehead imputes to a value of less than 0.3 a society that can be considered rather homogeneous (displayed in a sociogram, the individuals forming the nodes are on average all well connected to the others); to a value between 0.5 and 2.0 well differentiated societies (sub-units of individuals start to clump together well); and to a value higher than 2.0 extremely differentiated societies [23], [25].

To infer the change in the social differentiation of the groups across the conditions of proximity, affiliative behaviours' exchange and cooperation, I compared the social differentiations with (c) standard errors calculated via bootstrapping 10,000 random replicate matrices of the collected data. The first matrix produced, showed the preferred associations of monkeys found in space. This network carried the identity of each monkey with the one of its nearest neighbour, as long as this latter monkey was estimated within a maximum distance of 10 meters from the former. In this case, to avoid the spurious influence of the artificial food offered, both these individuals had to be further than 10 meters radius from the feeders. The second network was formed by the interactions of partners engaged in allogrooming, contact sitting and social play both during the training and cooperation phases. This matrix measures preferred and recurring partners exchanging affiliative behaviours. The third network was formed by behavioural interactions of individuals cooperating at the feeders. These interactions consisted in simply coming together to the feeders and sharing food.

ii) I made use of tests for preferred/avoided associations [23] to test how individuals associate for cooperating at the feeders. These tests compare the real matrices formed by the occurrences of cooperators at each experimental session in repetition with 10,000 randomly generated matrices of dyads or more individuals shuffled (variation of [26] by [23]), keeping as a constant their actual presence in the nearest surroundings during the experimental sessions. If an individual could not be found that day in the surroundings of the feeders, then I would not include that individual in the permuted matrix. In the text that follows and in the legends for figures and tables, I specify the permutations with the adjective ‘semi-random’ which represents the non-complete random shuffling of the individuals.

iii) Thereafter, I constructed Mantel Z-statistics models for each group. These tests were used to investigate cooperation patterns depending on individuals' attributes (sex, age, rank, and relatedness).

The same Mantel analyses were performed on feeding proximity occurrences. This was done to see whether these proximity data could predict preferred partnership during the experimental phase. These proximity data were collected during scan samples taken during the training phase comprising foraging behaviours from natural food sources of nearest neighbours.

At each comparison, the Mantel tests calculate whether there is a linear relationship between the cooperation formed by partners, whose reciprocal interactions are summarised in a matrix, and 10,000 of other permuted matrices of semi-random, dummy cooperation events. The correlation between the matrices was tested only on that part of the dataset that included the cooperation between the individuals of the smaller class (operating the black feeders) and the individuals of the larger class (white feeders). This was done not to bias the result with non-relevant cooperation events taking place between the fractions of individuals belonging to the same class of cooperators (when more than 2 individuals were then cooperating at the same time). In the Result section I provide, in addition, the matrix correlation coefficients (MCC), a descriptive measure of correlation between non-diagonal elements of the test matrices.

Linear mixed effect modelling was performed with SPSS 19. Network analysis and all related statistics were performed with SOCPROG 2.4 [25].

## Results

### (a) The Groups' Social Differentiation

To interpret the gradual social change in the groups' differentiation structure across the conditions of proximity, affiliative behaviours' exchange and cooperation, I compared the three social differentiation estimates for each group. Their standard errors were calculated via bootstrapping. The social differentiation estimates for the three groups are reported in Table 1, together with the relative standard errors and other parameters of accuracy.

**Table 1:** Values of social differentiation of the three groups according to the three conditions of (1) proximity in space of the nearest neighbour individuals within 10 m distance from each other, (2) between partners' display of affiliative behaviours of allogrooming, contact sitting and social play, and (3) display of the cooperative behaviour at the feeders.

Group	Condition	Individuals	Mean individuals identified per sampling period	Sampling period (days)	Number of associations or interactions	Social differentiation	SE
	Proximity	10	9.59	46	4281	0.3680	0.0340
**Picnic**	Affiliative interactions	10	9.13	33	2313	0.4145	0.0655
	Cooperation	7	6.35	20	763	0.5260	0.0740
	Proximity	18	16.14	51	5457	0.5110	0.0290
**Donga**	Affiliative interactions	18	13.97	51	3161	0.8650	0.0510
	Cooperation	13	7.20	25	784	1.6390	0.1160
	Proximity	17	12.13	31	1468	0.9630	0.0480
**Bay**	Affiliative interactions	18	10.36	28	930	1.0040	0.0910
	Cooperation	9	6.38	13	284	1.2770	0.0940

**Figure 2:**
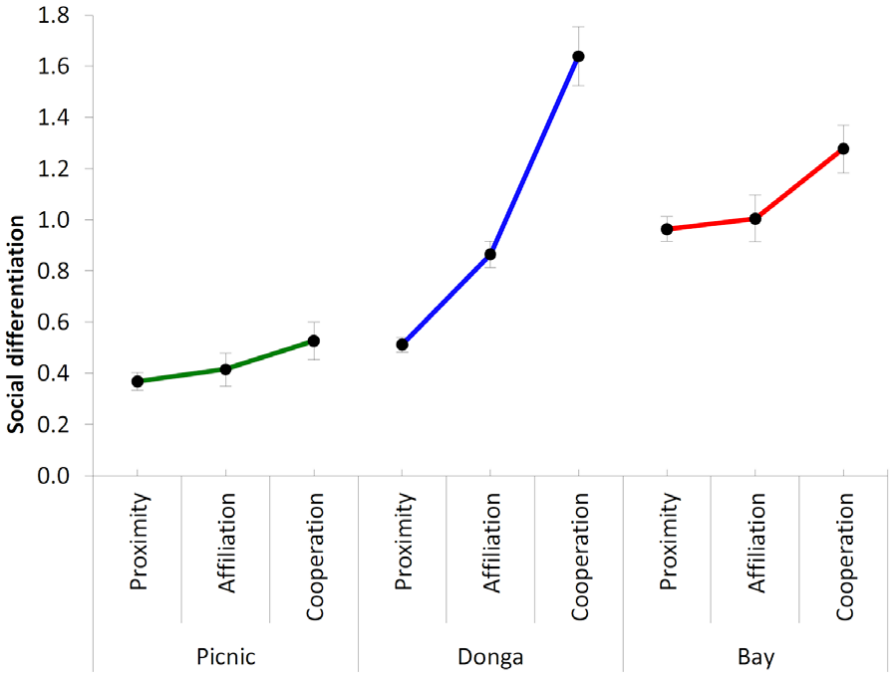
The social differentiation of the three vervet groups across conditions. For each group the social differentiation estimate was extracted during both training and cooperation from: (1) proximity distances of nearest neighbour individuals not at the feeders collected during scan intervals; (2) affiliative interactions of allogrooming, contact sitting and social play among individuals not at the feeders recorded on an all-occurrence basis; and (3) all-occurrence recordings of cooperation attempts from dyads or more individuals operating the feeders. Standard errors were calculated with bootstrapping procedure permuting 10,000 semi-random replicates of each type of matrix data from associating individuals. The dataset plotted in this graph is reported in full in Table 1.

The standard errors of the social differentiation indexes were calculated via bootstrapping, with 10,000 semi-random permutations. The social differentiation values with their standard errors have been plotted in Figure 2.

All the three groups showed a tendency of increase in the social differentiation when looking at proximity in space as compared to the exchange of social behaviours. Social exchanges occurred on average with a lower number of preferred companions than the frequency of meeting other individuals at least within 10 meters distance. A more significant result was the one provided by the comparison of the social differentiation indexes of proximity associations and affiliative interactions together, with the social differentiation value of cooperation. This result may be partly induced by the experimental design with the individuals of different classes having to join for cooperating. Still, all the three groups, when challenged with the cooperation experiment, reduced the number of partners (as witnessed by the increase of social differentiation, Figure 2 and Table 1). This result provided an indication that the process of selection of partners for sharing food to cooperate with was stricter than the one for sharing the same space and for exchanging social behaviours.

### (b) Pattern of association preferences

An initial analysis that shows how the individuals increased their selective choice for cooperating is reported in Supporting Information S1.

Applying a preliminary test for preferred/avoided associations (variation of [26] by [23]), I rejected the null hypothesis that individuals associate randomly for cooperating at the feeders. The Picnic group showed a real association index of 9.0, s.d. = 7.615, significantly different (p≤0.001) from a random, permuted association index of 12.34, s.d. = 7.517. Similarly, the Donga group displayed a real association index of 5.893, s.d. = 7.289, significantly different (p≤0.001) to a random association index of 7.045. The individuals of the Bay group did not (p = 0.001) associated randomly either (association index of 6.469, s.d. = 3.193) but gave a real association index of 4.714, s.d. = 4.286.

These tests suggest that there may be an underlying pattern of cooperation of preferred cooperation partners. I therefore tested my observation in this direction. In Supporting Information S1 I report results which show that the cooperation pattern within and between classes is different across the three groups. Finally, to find out whether the individuals' partner choice was dependent on intrinsic characteristics of the individuals preferring to share food together, I performed a partner choice analysis.

### (c) Social units of cooperative partners

**Figure 3:**
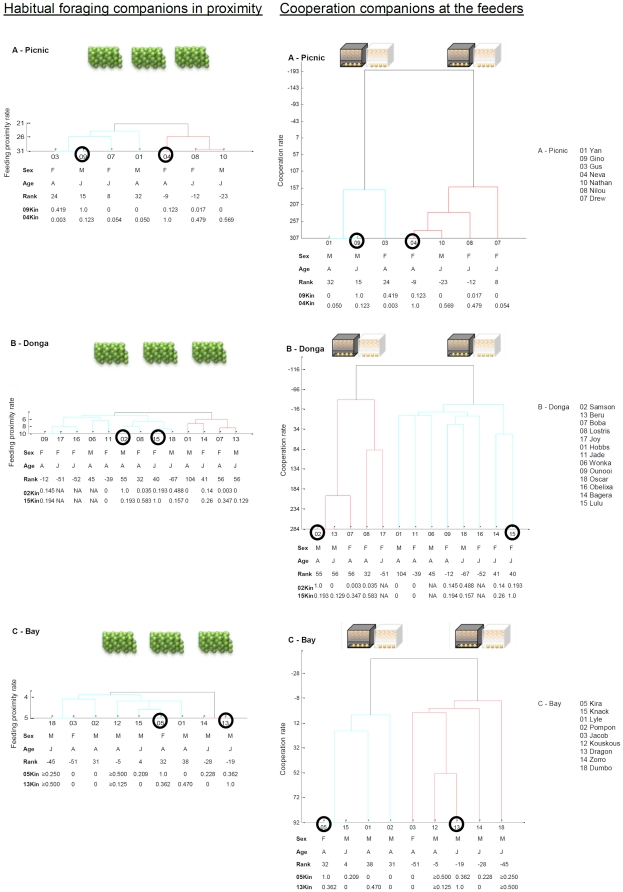
Dendograms of the social clusters of the three groups of wild vervet monkeys. The diagrams (inferred from cluster analysis using the Ward linkage coefficient) show the sub-units of companions during habitual foraging from natural sources and companions cooperating at the feeders during the experiment. Differently than during the habitual foraging activity, the monkeys discriminated and chose their cooperation companions at a higher rate. The clusters of preferred cooperation partners are more distinct during cooperation. The different colours (light blue and red) are assigned to the clusters by using the method of the modularity of Newman [34]. This method assigns the same colour to the clusters including the individuals who preferentially clumped together. Set the summed cooperation rates of the different individuals, the individuals' clustering is calculated by the difference between the observed and expected proportion of the total cooperation rates (y-axis). The probability of finding partners of different clusters interacting during cooperation is lower during cooperation. The feeding and cooperation rates on the y-axes were calculated by the sum of all cooperation attempts among individuals sharing food resources. The individuals marked with a black circle represent the smaller cooperator class able to operate the feeders in combination with at least one other member of the larger class (all the remaining individuals of each group). For the Picnic and Bay group (A and B), each one of these individuals was found most of the times in combination with a subset of preferred partners (either light blue or red coloured clusters). In the Donga group (C), this did not happen as distinctly (interaction rates of individual 15 proximate to 0) because of the discussed relatively high-ranking position of individual 15. On the x-axes the individuals are tagged with their sex, age class of whether adults or juveniles, dominance rank estimated with the David's Score (rounded to its closest integer, see Supporting Information S1 for further description), and the relatedness coefficient of Queller & Goodnight. The relatedness coefficients reported refer only to the relations of the two individuals of the smaller class with all the others of the larger class. A 0.5 coefficient means first order generation (e.g. son), 0.25 is relatedness at second order (grandson). The coefficients of three individuals from the Donga group could not be reliably extracted and are therefore not available, missing as well the relatedness of two individuals from the Bay group (id 12 and 18); I was able to partially infer them through the known maternity link and from genotyped siblings (see Supporting Information S1 for further description). The Ward's linkage method used to build the clusters can bear negative values of the ordinate as it uses the increase in the total within-cluster sum of squares because of joining two clusters at a time (the within-cluster sum of squares is defined as the sum of the squares of the distances between all objects in the cluster and the centroid of the cluster). According to the extracted cophenetic coefficients, the two A and C dendograms give a faithful representation of the social structure of the three groups: 0.97 for A and 0.79 for C. The social representation of the monkey group B is less faithful to reality, with a coefficient of 0.62. A cophenetic coefficient of 1.0 means a perfect fit of the dendogram with the data and 0.8 is generally taken as good estimate [23].

Two social units of cooperating individuals split from each of the three groups. The two members of the smaller class gathered around themselves other individuals from the larger (Figure 3). The preferred partners of each subunit did not mix with individuals of the other subunit. This was shown by the very low cooperation rates at which the two subunits of individuals cooperated with each other (Figure 3, Cooperation phase as opposed to Habitual foraging). The two subunits clumped around the two individuals of the smaller class indicating (together with the following partner choice analysis) that the larger class members did not switch between individuals at the black feeders.

For the Donga group, the two social units arising from the feeding experiment were less distinct (lower cophenetic correlation coefficient of 0.62 for the Donga then the other two groups of 0.97 for Picnic and 0.79 for Bay, calculated from cluster analysis, Figure 3). This was probably due to the fact that in this Donga group the two members of the smaller class of cooperators belonged both to the higher ranking individuals (individual 02 presenting dominance indices of +55 David's Scores and individual 15 with David's Scores of +40. The second individual was chosen to belong still to the smaller class after her lower dominant sister that was chosen at first disappeared from the group). This group seems therefore to differentiate less than the other groups.

### (d) Partner choice

The cluster analysis of Figure 3 shows the subunits of partners cooperating at the feeders (cladograms on the right side). Compared to habitual foraging, the Picnic group maintained in general the same preferred partners during the two conditions. Only individual number 07, a juvenile female, changed preferred partners. The Donga group in general did not conserve the preferred associating partners between normal foraging and feeding at the feeders. Also in the Bay group, in general, preferred foraging partners did not conserve their preferred association during cooperation. The (less defined) cluster formed by two juvenile male partners foraging often together became more distinct during cooperation including also other subordinate individuals.

To investigate the causing factors for the occurrence of non-random cooperation, I looked at whether there was a correlation between the recurring cooperators and their identity in terms of sex, age class, rank, and relatedness. In addition, relatedness was also tested, controlled at the same time for the matriline and sibling strains. Since the matriline is generally known to the monkeys, this control allowed to test whether relatedness is taken into account by the subjects outside the members of the same matriline. The same analyses were performed on feeding proximity occurrences to check whether they could predict food sharing during cooperation. The Mantel Z-tests are reported in and they show the correlation between the matrices formed by dyads or more individual cooperating and their sex, age, rank and relatedness type.

During normal foraging, the individuals cooperated without a given pattern choice of same or different sex attribute, age class, rank, or relatedness. On the other hand, a specific trend appeared during the cooperation condition. During this phase, males cooperated with females indiscriminately and vice versa (Table 2). This holds true in the Picnic and the Donga, but not in the Bay group where, because of a large predominance of males, a same-sex preference was found. In the three groups, adults cooperated indiscriminately with juveniles, and juveniles with adults, except in the Picnic group where a mixed sorting was found. Conversely, in all the three groups, higher ranking individuals cooperated consistently more with other higher ranking individuals, and lower ranking individuals with other lower ranking individuals (Table 2).

**Table 2:** Multiple matrix analyses from feeding proximity and cooperation interactions of the three groups with their members' identity in terms of sex, age class, rank, and relatedness.

			Feeding proximity		Cooperation	
Group	Identity	Individuals of the smaller/larger class	Mantel Z-test p-values	Matrix Correlation of Mantel tests	Mantel Z-test p-values	Matrix Correlation of Mantel tests
**Picnic**	Sex	2/5	0.896	−0.650	0.493	0.161
	Age class	2/5	0.902	−0.382	0.999	−0.976
	Rank	2/5	0.114	0.531	**0.041**	0.685
	Relatedness	2/5	0.999	−0.627	0.853	−0.514
	Relatedness controlling for matriline and siblings	2/5	0.896	−0.308	0.455	−0.017
**Donga**	Sex	2/11	0.914	−0.097	0.695	−0.224
	Age class	2/11	0.651	−0.097	0.510	0.038
	Rank	2/11	0.630	−0.040	**0.045**	0.323
	Relatedness	2/7	0.352	−0.093	0.091	0.462
	Relatedness controlling for matriline and siblings	2/7	0.317	−0.012	0.156	0.370
**Bay**	Sex	2/7	0.999	0	0.999	−0.267
	Age class	2/7	0.665	−0.098	0.348	0.131
	Rank	2/7	0.283	0.202	**0.043**	0.538
	Relatedness	2/7	0.227	0.257	0.273	0.173
	Relatedness controlling for matriline and siblings	2/7	0.146	0.343	0.233	0.207

In addition, the cooperation interactions were further compared to the relatedness controlling the former for matriline and sibling identity apparent to the monkeys. The relatedness coefficients of three individuals from the Donga group are missing, and two from the Bay were partially inferred through the known maternity link and deducing them from fingerprinted siblings. The tests were performed between the mixed cooperator classes and the total number of individuals of each class is displayed. Even though during normal foraging activity the monkeys it was not imposed any class distinction, in order to compare the two conditions, the class distinction was also imposed to these normal behaviours excluding interactions from same class partners. Mantel Z-tests are reported together with their matrix correlation coefficients (the correlation between non-diagonal elements of the test matrices). The p-values significant are bold typed.

Was this due to genetic similarities, given the small size of the groups? One would expect matriarchal vervet individuals that are related, also to bear similar dominance index, leading to a correlation between rank preference and genetic similarity. Although individuals belonging to the same matriline tended to stand on similar dominance positions, I did not find the null hypothesis of cooperation among kin individuals to be met. The individuals of the three groups cooperated irrespective of their relatedness. Although the limited genetic variability found in these monkeys often belonging to few matrilines within each group, I did not find a tendency of kin partners to share food (with a p-values that would have gradually moved from the random value of 0.5 to the related one of 1 – Table 2). In contrast, the two groups of the Donga and the Bay gave values tending towards the remarkable conclusion of preference for matching unrelated partners. The occurring partners at the feeders were thus more often coming from more distantly related family lineages, at least limiting the genetic relatedness analysis to the two classes of cooperators. This finding was not as strong as to provide significant p-values at a 0.05 significance level. All specific p-values of the models testing for partner preference are found in Table 2.

## Discussion

The current study shed light on the modified social dynamics that arose in three wild primate groups when an experiment to elicit cooperation was set up in the field. The limited and patchy resources were offered to couples or multiple monkeys, side by side, operating a food releasing mechanism.

Firstly, the monkeys did succeed cooperating with other individuals. The partners in fact adapted to the sharing food condition by becoming able to cooperate (more over time, as shown in Supporting Information S1). I therefore demonstrate that vervets can in general cooperate in the field.

Due to the cooperation condition, the individuals congregated together more heterogeneously when co-feeding. Thus cooperation increased the groups' social segregation tendency. Associations of proximity distances and interactions of affiliative behaviours exchanged before the experiment did not predict the interaction pattern during cooperation. That animals and humans cooperate with preferred partners is not a new element in the literature (e.g. [27], [28], [29]). What is new here is that social networks previous to cooperation did not predict occurring ones during cooperation. What we found is an indication that the process of selection of partners to cooperate with became stricter than the one for sharing the same area (up to 10 m apart), or for exchanging affiliative behaviours. This result could be explained by the availability of possible partners to match with at the feeders and individuals' preferences for matching (as in a matching market, [17], with limited number of partners joining together). Providing the monkeys with limited and patchy resources caused agonism at the feeders. It is therefore possible that some individuals opted to approach the feeding resources when preferred partners were present and avoided approaching at other times not to get involved into conflicts with other group members. Hence the three groups of vervets moved from presenting rather homogeneous societies to increasing their social differentiation and becoming more segregated when cooperating. The prediction of a resident-nepotistic strategy, in which rank differences are strongly enforced [30] was therefore met in an artificial setup as this one.

With the help of network analysis (of particular interest in primate behaviour studies, [31] and [32]) I could quantify the social differentiation of group across different conditions.

With these field experiments I was able to show that monkeys cooperate at the feeders choosing specific preferred partners. The preferred partner combinations did not tend to change during following experimental sessions. In fact dyads or multiple individuals were observed consistently at the feeders as shown by their consistently repeated cooperation rates.

To check for the reason of preferred sub-units of individuals, I tested multiple variables describing the status of each monkey within each group. I thus constructed models to test sex, age class, rank, and relatedness as affinity for partner choice. Across the three groups, the monkeys preferred cooperating at the feeders in arbitrary combinations of sex and age class. Surprisingly, I did not find that the monkeys preferred to share food with related individuals. Significantly, I found a consistent discriminant of dominance of the cooperators. Dominant individuals preferred cooperating with other dominants and subordinates with other subordinates.

Recently Jaeggi and colleagues [33] have shown the importance of rank in the context of reciprocal food sharing. This study, however, was done in captive chimps and bonobos and without the constrained cooperation condition enforced.

My result suggests the key role that social rank has in vervet monkeys, when constrained to access and share limited resources in a limited space. These primates showed heterogeneous social networks and rank-related nepotistic behaviours which prevented individuals of very different rank statuses mixing together for cooperating. The strategy applied by the test subjects may be an evolutionary stable one. If we assume that it is convenient to avoid conflicts between higher and lower ranking individuals, these vervets seem avoiding mixing these two rank categories as to circumvent conflicts for accessing and sharing food together.

The effect of rank on cooperation may be also justified in terms of tolerance: dominants tolerate more other dominants and subordinates other subordinates.

Finally, looking at kinship, these monkeys did not show an association trend confirming the common theory that related individuals would preferentially support each other in cooperation [11]. These study groups would have been likely candidates for showing cooperation among kin individuals, given the limited genetic variability in these small groups. Nevertheless this expectation was not met, and the monkeys cooperated irrespective of their relatedness, with a tendency to find partners from a different family.

Most significantly we see that dominance status plays a key role in this augmented social differentiation and gets exacerbated under a condition with two cooperation classes. It can be argued that the division of the groups into two classes of skewed size causes the clumping of the individuals around the two individuals of the smaller class. I chose the size of the smaller class to be as small as comprising two individuals to test whether the smaller class gets rewarded, after the experiment, for its influential commitment in cooperation (Pansini et al, in prep.). The reduced class size does not explain however why partner choice was attained with determined partners so strictly and no exchanges occurred throughout the cooperation phase.

In summary, monkey partners preferred to cooperate with other partners of similar rank status.

In order to test whether cooperation induces other social groups to differentiate, I recommend the implementation of this experiment in other primate species and mammals.

## Supporting Information

Supporting Information S1 (DOC)

Video S1 (AVI)
