# Supplementary material for: Induced Cooperation to Access a Shareable Reward Increases the Hierarchical Segregation of Wild Vervet Monkeys
Source: PLoS One. 2011 Jul 20;6(7):e21993. doi: 10.1371/journal.pone.0021993 (PMC3140478; doi:10.1371/journal.pone.0021993)
Supplement: Supporting Information S1 — (DOC) [file pone.0021993.s001.doc]

# Online Supplementary material

## Supplementary Methods

### Data collection

For the Picnic group, I collected training and cooperation data from May to July 2008 and from September to November 2008. From the other two groups of the Donga and the Bay, the data were collected at the same time on rotated days from May to September 2009. The training phase stretched for 26 sessions with the Picnic group, 26 sessions with the Donga, and 18 with the Bay. The cooperation phase instead was repeated during 20 sessions with the Picnic, 25 sessions with the Donga, and 13 sessions with the Bay.

### Feeding Protocol

During an initial pilot feeding test, I checked which food items were preferred by the monkeys. I chose to use toasted rice cereal (even if different from some more natural fruit items) because of its low specific weight, large volume, and resulting low caloric impact (3.9 cal/gr). The feeders were designed in a way to dispense small food provisions. Their volume was 0.0035 m3each and their size 20x20x13 cm.

To avoid over-habituation to the food type and to limit the impact on their natural behaviour, I tended not offering artificial food to the same group on successive days. Even if the food offered was appreciated by the monkeys, the experiment did not have a serious impact on their natural feeding habits. At turns, when not having access to the feeders, they used to forage from the trees and on the ground, throughout the day and before, during and after the experimental sessions. At each button release attempt a handful of toasted rice cereal was dispensed. The monkeys became habituated to access food several times during an experimental session (see Video 1 for an example of the subjects feeding during the cooperation phase).

On average, each experimental session lasted three hours, during which several monkey partners could attempt to operate the feeders both on their own (during the training phase) and share food (during the cooperation phase).

During the training phase of the experiment the individuals of each of the three groups were divided in two classes. For the smaller class (the one comprised of two individuals), in the Picnic group one individual was a juvenile male higher ranking, and the other was an adult female lower ranking. In the Donga, one adult male higher ranking, and one juvenile female lower ranking. In the Bay group, one adult female higher ranking, and one juvenile male lower ranking. During the training phase, these white feeders were positioned in the same area at 2-3 meters distance from the blacks. The training phase terminated once all the individuals of the black class and at least half of the monkeys of the larger class became able to use the feeders of the right colour. I assessed this ability as accomplished once each of the trained monkeys could independently activate the food releasing mechanism.

During the cooperation phase, the total number of feeders positioned on the ground was 4; that is to say two joint feeders at 2-3 meters distance from each other (Error: Reference source not found). By the use of remote controls, one observer (R. Pansini) could activate or deactivate the triggers and hence allow the right combination of monkey classes to feed (if the monkeys were pressing the activated trigger on their own). With the monkeys releasing the triggers, we can specify cooperation as a work of active food producing (same emphasis as in the 'active' food sharing of ). In fact, by operating the buttons the monkeys were releasing the food reward on their own. The operable triggers on the joined feeders were two (one on top of each single box) and the individuals feeding were dyads or more individuals. So as long as one monkey was activating one trigger, both feeders were providing food to all in front. The previous training phase had a long lasting effect on the monkeys and the individuals of the smaller class were still operating the triggers of the black feeders, and those of the larger the white feeders.

### Estimation of Individuals’ Hierarchy

The hierarchical rank of the monkeys was computed by examining submissive behaviours at the feeders. They consisted of a ‘looking-away’ or a ‘walking-away’ behaviour, displayed to avoid the individual that would approach and/or take the position of the submitted individual. These behaviours were collected during the training phase of the experiment, as a result of agonism within 5 m radius from the boxes, and when the feeders were not joined.

Rank values were assigned in the form of David’s Scores  which calculate cardinal, non-linear rank indices for each individual. Thanks to these scores I can quantify, and not just order, the differences in ranks among all individuals participating in contests. The scores are based on an unweighted and weighted sums of the individuals’ dyadic proportions of wins, combined with unweighted and weighted sums of their dyadic proportions of losses . The scores provide a heavier weight to winning a contest with a high-ranking animal compared to defeating a low-ranking animal. This induces known advantages that make them robust descriptors of unbalanced contest data. With these scores, the higher ranking individuals tend to bear positive coefficients, whereas lower ranking have negative values. I also calculated an alternative to the David’s scores, the Normalized David’s Scores (no statistics shown here). These latter were recently shown as suitable measures in case of contests around feeding resources and they confirmed the output of the statistics of the David’s Scores (reported in the Result section). The rank scores were obtained with the MATLAB programs SOCPROG 2.4 .

### Estimation of Relatedness

Coefficients of maternal relatedness of most of these monkeys were known from demographic records thanks to previous studies . To establish full relatedness including patrilines of both juveniles and adults, genetic analysis was performed from faecal samples. Forty-three faecal samples from individuals of the three groups were collected in parallel with the behavioural observations. An average of two samples per animal was taken preferably during the dry season, so to aim at finding a higher concentration of intestine cells in the specimens. Directly after defecation, the faecal samples were preserved in RNAlater solution and a copy set in ethanol. Those in ethanol were desiccated three days later with silicagel for their longer preservation in time and subsequent lab analyses .

Relatedness was measured among all pairs of individuals coming from the comprehensive pool of collected data of the three groups. A comprehensive matrix bearing a total of 42 monkeys was therefore produced with 903 extracted pairwise genetic distance coefficients. The relatedness estimator used was the one of Queller & Goodnight based in this case on 13 microsatellite loci. I double checked the results with the Wang estimator which confirmed consistency and robustness of the Queller & Goodnight genetic distances reported in results.

The relatedness coefficients of three individuals from the Donga group could not be reliably extracted and are therefore not available. For the statistics, I was still able to infer the coefficients of two individuals from the Bay group through the known maternity lineage and from genotyped siblings in common. Only two of these three individuals shared food (Error: Reference source not foundC). Their relatedness indexes bear a ‘≥’ symbol, meaning the estimated minimum relatedness coefficient with the individuals of the smaller class of cooperators. The value could be higher than the one shown in case the father(s) of these monkeys is (are) inbred with the mothers.

### Increase in Cooperation Events

I made use of a linear mixed effect model to test whether the amount of cooperation events at feeders changed over the course of the cooperation phase across the three groups. Confidence intervals were kept at a 95% level for the standard deviation and residuals were checked for normality by plotting a q-q plot. The p-value reported originates from the minimal model (as indicated by lower AIC indexes) produced applying an autoregressive moving average covariance to the time series cooperation trials. I fitted this covariance structure given the longitudinal and repeated pattern of the experiment, iterated in consecutive days.

### Social Differentiation

Association and interaction data were used to structure the networks and to infer (a) social differentiation indexes, (b) affiliation and cooperation rate indexes, and (c) standard errors of social differentiations.

Having collected the identity and proximity measure of individuals at within 10 meters proximity and when exchanging social behaviours (both affiliative interactions and cooperation), I extracted (a) social differentiation indexes from these three networks (plotted in Figure 1.

To perform statistical analyses, it was necessary to extract (b) interaction rate indexes which sum up the matrices of the repeated measures of relationships of dyads of individuals collected at each sampling session. The interaction rate index, used to calculate matrices of individuals associating among each other, is given by the sum of all the social exchanges among the individuals. This index estimates the proportional frequency of time each individual spend associated with somebody else .

The social differentiations were compared with (c) standard errors calculated via bootstrapping 10,000 random replicate matrices of the collected data. The bootstrapping procedure provides the most accurate method to date to obtain standard errors , as compared to those acquired from F-statistics. This procedure also allows a cross-comparison of the data taken at different time intervals (scan and all-occurrence samples).

### Within and Between Classes Variation

In another analysis, I went deeper studying what happened during cooperation. I tested whether the interaction rates of cooperation events between and within the two classes of cooperators were different. The idea behind is to show whether interactions between classes, those of interest here, are different than interactions within the same class, usually across members of the larger class. As explained, during cooperation co-feeding could not take place if not both class members were present. During multiple-partner cooperation events, usually one monkey from the smaller class would feed with others, at least two or more members of the larger class. Mantel t-tests with 10,000 permutations were used to assess this null hypothesis of similar cooperative events across classes .

## Supplementary Results

A summary with the total data collected from the three groups is reported in Table 1. In there, are listed (1) the number of individuals followed, (2) the sampling sessions in days, (3) the mean individuals identified per sampling session, and (4) the total number of associations or interactions.

### Natural foraging behaviour and nearest neighbour distances

I recorded instances of foraging behaviour in natural conditions from trees, lower vegetation and on the ground. I collected scan samples of these occurrences to assess habitual foraging companions and their distance in space. These data are the same as those reported in left cladograms of Figure 3 of the main text. The three groups showed similar recurring distances on average of foraging companions. These data included all individuals in the group, including dyads of mothers with the young offspring.

In the Picnic group the individuals foraged at 3.74 meters from each other (± 0.153) (N=213). In the Donga group the monkeys fed at 4.27 meters (± 0.212) (N=139). In the Bay at 4.48 meters (± 0.216) (N=129).

On the other hand, during the cooperation experiments, the individuals fed from the apparatuses at non-natural, adjacent position in space. This is an indication that the presence of the partners at such close distance is a manifestation of cooperation behaviour. Cooperation intended not as instrumental, but as task solving for being at such close distance in space. The distance was not manifested, on average, during normal foraging conditions.

### The Increase of Cooperation Events

In the Picnic group 7 out of 10 individuals ended up cooperating; in the Donga, 13 out of 19; and in the Bay 9 out of 17.

I was expecting the monkeys to gradually learn that the food reward could be accessed as long as the two cooperator classes stood in front of the feeders together.

With the iteration of the experiment trials in the condition of cooperation the monkeys gradually increased their cooperation attempts. As plotted in Figure S1, the number of these cooperation events increased in frequency. This increase was found statistically significant when tested with a linear mixed model with the three groups included in the mixed model as random effects (estimate +0.205, SE = 0.040, t_3,58_ = 5.073, p = 0.038).

### Within and Between Classes Variation

After I tested if, during the cooperation phase, the interaction rates of cooperation events between and within the two classes of cooperators were different. By doing so, I studied also the difference between co-feeding attempts of dyads (one individual in front of the black feeder and the partner in front of the white) against larger combinations of monkeys (usually one individual from the smaller class and two, three or more from the larger in front of the white feeders). Results are expressed with a t-value (with infinite degrees of freedom), a p-value (for 1-tailed test) for the analytical approximation, a permutation p-value (with 10,000 permutations set), and a matrix correlation coefficient. For all the three groups, cooperation rates between and within the classes of individuals were statistically different (Mantel t-statistics: Picnic: t = -1.561, p < 0.0001, Matrix correlation (MC) = -0.314; Donga: t = -2.129, p < 0.0001, MC = -0.369; Bay: t = -2.761, p < 0.0001, MC = -0.499).

The social differentiation test and the within and between classes one suggest that the monkeys chose each other according to a specific pattern of partner choice. This was tested with the analyses of partner choice (both in the main text and thereafter here), which finds out how rank plays a determinant role in the partner choice.

## Supplementary References

## Supplementary Figures


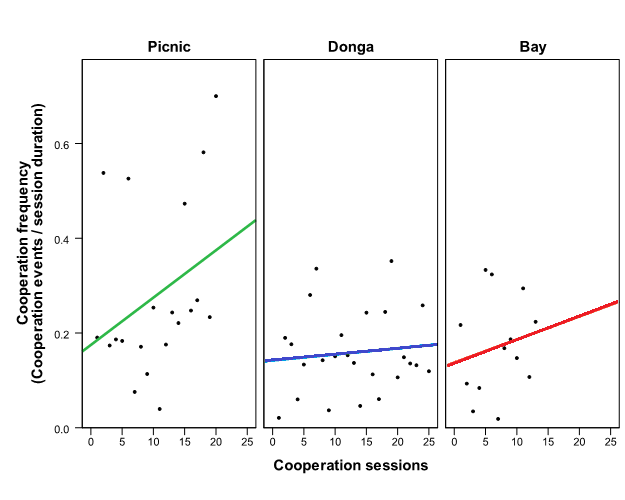


Figure S1 – The increase in cooperation events during the cooperation phase of the experiment. Dots represent total number of cooperation events by day divided by the duration of that experimental session in minutes. A linear interpolation line has been added to the data to show the increase of cooperation frequency at successive sessions. An experiment was considered as concluded when the monkeys would leave the experiment area after having fed for long enough.
